# Anti-plasmodial and insecticidal activities of the essential oils of aromatic plants growing in the Mediterranean area

**DOI:** 10.1186/1475-2875-11-219

**Published:** 2012-07-02

**Authors:** Mario Dell’Agli, Cinzia Sanna, Patrizia Rubiolo, Nicoletta Basilico, Elisa Colombo, Maria M Scaltrito, Mamadou Ousmane Ndiath, Luca Maccarone, Donatella Taramelli, Carlo Bicchi, Mauro Ballero, Enrica Bosisio

**Affiliations:** 1 Dipartimento di Scienze Farmacologiche e Biomolecolari, Università degli Studi di Milano, Milan, Italy; 2 Co.S.Me.Se, Dipartimento di Scienze della Vita e dell’Ambiente, Macrosezione Botanica e Orto botanico, Università degli Studi di Cagliari, Cagliari, Italy; 3 Dipartimento di Scienza e Tecnologia del Farmaco, Università degli Studi di Torino, Torino, Italy; 4 Dipartimento di Scienze biomediche, chirurgiche e odontoiatriche, Università degli Studi di Milano, Milan, Italy; 5 Laboratoire de Biologie Moléculaire, UMR 198, Paludologie IRD HANN, Dakar, Sénégal

**Keywords:** Essential oil, Insecticidal activity, *Plasmodium falciparum*, *Satureja thymbra*, *Myrtus communis*, *Thymus herba-barona*, Thymol, *Anopheles gambiae*

## Abstract

**Background:**

Sardinia is a Mediterranean area endemic for malaria up to the last century. During a screening study to evaluate the anti-plasmodial activity of some aromatic plants traditionally used in Sardinia, *Myrtus communis* (myrtle, Myrtaceae), *Satureja thymbra* (savory, Lamiaceae), and *Thymus herba-barona* (caraway thyme, Lamiaceae) were collected in three vegetative periods: before, during and after flowering.

**Methods:**

The essential oils were obtained by steam distillation, fractionated by silica gel column chromatography and analysed by GC-FID-MS. Total oil and three main fractions were tested on D10 and W2 strains of *Plasmodium falciparum in vitro*. Larvicidal and adulticidal activities were tested on *Anopheles gambiae* susceptible strains.

**Results:**

The essential oil of savory, rich in thymol, was the most effective against *P. falciparum* with an inhibitory activity independent from the time of collection (IC_50_ 17–26 μg/ml on D10 and 9–11 μg/ml on W2). Upon fractionation, fraction 1 was enriched in mono-sesquiterpenoid hydrocarbons; fraction 2 in thymol (73-83%); and fraction 3 contained thymol, carvacrol and terpinen-4-ol, with a different composition depending on the time of collection. Thymol-enriched fractions were the most active on both strains (IC_50_ 20–22 μg/ml on D10 and 8–10 μg/ml on W2) and thymol was confirmed as mainly responsible for this activity (IC_50_ 19.7± 3.0 and 10.6 ± 2.0 μg/ml on D10 and W2, respectively). The essential oil of *S. thymbra* L. showed also larvicidal and adulticidal activities. The larvicidal activity, expressed as LC_50_, was 0.15 ± 0.002; 0.21 ± 0.13; and 0.15 ± 0.09 μg/ml (mean ± sd) depending on the time of collection: before, during and after flowering, respectively.

**Conclusions:**

This study provides evidence for the use of essential oils for treating malaria and fighting the vector at both the larval and adult stages. These findings open the possibility for further investigation aimed at the isolation of natural products with anti-parasitic properties.

## Background

Malaria, with AIDS and tuberculosis, is one of the three major communicable diseases linked to poverty. The unavailability of a vaccine and the spread and intensification of drug resistance over the past 15–20 years have led to a dramatic decline in the efficacy of the most affordable anti-malarial drugs. Because of their importance to humans since ancient times, plants have been the object of relevant scientific investigations to identify bioactive anti-plasmodial compounds [1], a view supported by the fact that natural products have become effective drugs against malaria.

In addition to anti-malarial drugs, plant constituents are of interest for application as insecticides and repellents. Among plant-derived compounds, essential oils (EOs) received great attention due to the variety of their biological activities including anti-bacterial, fungicidal, pesticidal, and anti-malarial [2]. Most of plant-based insecticides and repellents are derived from plants containing EOs. Receptors responding to DEET (N, N-diethyl-3-methylbenzamide), also respond to thujone, eucalyptol and linalool [3].

Sardinia is a region of Italy endemic for malaria until the first half of the past century. The infection was counteracted through the use of autochthonous plants as febrifuge or mosquito control agents. This Mediterranean island is rich in aromatic plants used for their content in EOs. Plants in the Sardinian flora include *Myrtus communis* (myrtle, Myrtaceae), *Satureja thymbra* (savory, Lamiaceae), and *Thymus herba-barona* (caraway thyme, Lamiaceae) [4].

A decoction of myrtle was in use for the treatment of malarial fevers. The EOs of several plants including myrtle showed anti-plasmodial activity towards various strains of *Plasmodium falciparum*[1,5].

Myrtle is a potent anti-bacterial and may work as an immune-stimulant in fighting cold, influenza or infectious diseases. Recent reports have described the anti-oxidant [6] and anti-inflammatory [7] activities of myrtle extracts. Oligomeric acylphloroglucinols were isolated from myrtle leaves and showed potent multi-drug resistant anti-bacterial effects [8,9]. Myrtucommulone, one of the acylphloroglucinols, inhibited Plasmodium growth in a nanomolar range [8], and caused a striking reduction of parasitaemia in mice infected with *Plasmodium berghei*[10].

Savory is a perennial aromatic shrub growing in the East Mediterranean basin from Turkey to Greece and Libya. In Italy, it grows only in Southern Sardinia. Previous studies reported a larvicidal activity by the EO of *S. thymbra* against the larvae of *Drosophila melanogaster*, and *Culex pipiens*, the vector responsible for the transmission of West-Nile virus, filariasis and Japanese encephalitis [11]. The EO obtained from this plant also showed an acaricidal activity [12]. The EO of myrtle and savory showed insecticidal activity against adults of the Mediterranean flour moth *Ephesta kuehniella*, the Indian meal moth *Plodia interpunctella* and the bean weevil *Acanthoscelides obtectus*[13].

Caraway thyme is a perennial plant endemic in Majorca, Corsica and Sardinia [14]. The EO of caraway thyme showed larvicidal effects on *Limantria dispar*, one of the most serious pests of cork oak forests [15]. The EOs of *Thymus spp* (*T*. *transpicatus,* and *T. vulgaris*) and *Myrtus communis* showed larvicidal activity against *Anopheles*[16-19]. *Thymus spp* (*vulgaris* and *serpyllus*) and *Myrtus communis* were shown to be repellent against *Aedes, Anopheles* and *Culex* mosquitoes [20,21]. Thyme EO is patented for anophelifuge activity [22].

In view of the increasing interest in developing new anti-malarials or insecticides of natural origin as an alternative to chemical insecticides, this study selected plant species from the Sardinia flora traditionally used to combat malaria. The EOs of *S. thymbra*, *M. communis*, and *T. herba-barona* were obtained and tested for anti-plasmodial activity against *P. falciparum*. The EO of *S. thymbra* was also tested for adulticidal/larvicidal activity against *Anopheles gambiae*.

## Methods

### Chemicals

Pure standard of α-pinene, β-pinene, myrcene, limonene, terpinen-4-ol, sabinene hydrate, thymol, germacrene D, caryophyllene oxide, were purchased from Sigma Aldrich (Milan, Italy). They were selected as representative of the different chemical classes of the essential oil components for the calculation of response factors towards an internal standard (n-decane) to correct the potential shift of flame ionization detector (FID) response towards the different classes of compounds [23]. Standard solutions at different concentrations of each of them containing 5 μg/μl of n-decane as internal standard were dissolved in cycloexane. Solvents were all HPLC grade from Riedel de Haen (Seelze, Germany).

### Plant material and essential oil preparation

Aerial parts of *M. communis*, *S. thymbra* and *T. herba-barona* were collected in Jerzu, Cagliari and Osini (Sardinia, Italy), respectively, in three different periods, before (sample A), during (sample B) and after flowering (sample C) to investigate how the variability of the EO could affect the biological activity.

They were botanically identified and registered with the specimen numbers 514 (*Myrtus*), 1,079 (*Satureja*) and 1,065 (*Thymus*) at the General Herbarium of Dipartimento di Scienze della Vita e dell’Ambiente, Macrosezione Botanica e Orto botanico, University of Cagliari (CAG). EOs from fresh plant materials were obtained in agreement with the European Pharmacopoeia VII ed.: fresh crushed aerial parts were submitted to steam distillation for five hours. The resulting EOs were left to stabilize for one hour.

Successively, each sample (A, B, C) of the essential oil of *S. thymbra* was fractionated on silica gel column chromatography using petroleum ether (PE) with increasing amount of ethyl acetate (EtOAc), giving seven fractions, three of them in significant amounts (fractions A1-A3, B1-B3, C1-C3, see Results).

### Essential oil analyses

GC analyses were carried out with a Thermo Electron TRACE GC Ultra (Rodano, Italy) provided with a FID detector. GC/MS analysis was carried out with a Thermo Electron TRACE GC Ultra coupled to a Trace DSQ mass spectrometer operating in Electron Impact mode.

GC-FID-MS analyses were carried out on a Mega5 column (5% phenyl methyl polysiloxane) 25 m, 0.25 mm i.d., 0.25 mm film thickness, from MEGA (Milan – Italy). GC and GC-MS conditions: injection mode: split; split ratio: 1: 20. Temperatures: injector: 220°C, transfer line: 230°C; ion source: 230°C; carrier gas: He, flow-rate: 1.0 ml/min in constant flow-mode. MS detector operated in electron impact ionization mode (EI) at 70 eV, scan rate was 1,111 amu/s and mass range of 35–350 m/z. Temperature program: from 50°C (1 min) to 220°C (5 min) at 3°C/min.

The components were identified by comparison of both their linear retention indices (*I*_s_^T^), calculated versus a C_8_-C_25_ hydrocarbon mixture, and their mass spectra to those of authentic samples or with data from the literature [24]. The FID response factors were used for the semi-quantitative determination of the volatile components.

### Anti-plasmodial activity

The chloroquine (CQ) -sensitive (D10) and the CQ-resistant (W2) strains of *P. falciparum* were sustained *in vitro* as described by Trager and Jensen [25]. Parasites were maintained at 5% haematocrit (human type A-positive red blood cells) in RPMI 1640 (EuroClone, Pero, Milan, Italy) medium with the addition of 1% AlbuMaxII (Invitrogen, Monza, Italy), 0.01% hypoxantine 20 mM Hepes (EuroClone, Pero, Milan, Italy), 2 mM glutamine (EuroClone, Pero, Milan, Italy). The viability and parasitaemia of cultured parasites was evaluated by light microscopy analysis of Giemsa-stained blood smears. The parasitaemia was maintained within 1% and 4% diluting the cultures with uninfected erythrocytes in complete medium at 5% haematocrit. All cultures were maintained at 37°C in a standard gas mixture consisting of 1% O_2_, 5% CO_2_, 94% N_2_.

The EOs and fractions were dissolved in DMSO and then diluted with a medium to achieve the required concentrations (final DMSO concentration <1%, which is non-toxic to the parasite). Asynchronous cultures with parasitaemia of 1–1.5% and 1% final haematocrit were aliquoted into the plates and incubated for 72 hrs at 37°C. Parasite growth was determined spectrophotometrically (OD: 650 nm) by measuring the activity of the parasite lactate dehydrogenase (pLDH), according to a modified version of Makler’s method in control and drug-treated cultures [26]. EOs and fractions were tested at 1–100 μg/ml. Anti-plasmodial activity is expressed as the 50% inhibitory concentrations (IC_50_), each IC_50_ value is the mean ± standard deviation (sd) of at least three separate experiments performed in duplicate. Chloroquine was used as positive reference compound at 0.8-100 ng/ml and 8–1000 ng/ml on D10 and W2 strains, respectively.

### Plasmepsin II inhibition assay

Pro-plasmepsin (Pro-PLM) II was prepared according to the procedure previously described [27], with slight modifications [28]. Protein was diluted to the final concentration of 0.5 mg/ml in 50% glycerol and stored at −20°C. Pro-PLM II was activated by addition of one tenth volume of 100 mM sodium acetate buffer pH 4.7 following by incubation at 37°C for 90 min. The enzyme activity of PLM II was evaluated spectrophotometrically at 300 nm by following the cleavage of the chromogenic substrate Lys-Glu-Phe-Val-Phe-NPhe-Ala-Leu-Lys (where NPhe is para-nitro-phenylalanine), as described [28].

*Satureja thymbra* EO was tested at 1–50 μg/ml dissolved in DMSO (final concentration <1% of the sample volume). IC_50_ values (mean ± sd of three experiments in triplicate) were obtained using Graph Pad Prism 4.

### Cytotoxicity

Cytotoxicity was evaluated in human neonatal dermal fibroblasts (Cell applications, inc. San Diego, CA, USA, cat number 106-05n). Cell proliferation was followed by the MTT (3-[4,5-dimethylthiazol-2-yl]-2,5-diphenyltetrazolium bromide) test. Fibroblasts (2 × 10^4^/ml) were grown in DMEM (Dulbecco’s modified Eagle’s medium) containing 10% foetal calf serum, 1% penicillin/streptomycin, and 1% L- glutamine. The compounds to be tested were added to culture medium dissolved in DMSO 48 hrs after plating. The final concentration of the vehicle in control and test medium was 0.1% of the incubation volume. The effect of compounds (10–100 μg/ml) on cell proliferation was assayed at 24–48 hrs after treatment. Each concentration was tested in triplicate in three separate experiments.

### Adulticidal activity

*Anopheles gambiae*, molecular form M, originated from Yaoundé, Cameroon and maintained since more than one decade under the conditions of the insectary in IRD Dakar Senegal (at temperature 28 ± 2°C and relative humidity 80% + −5%) were used [29]. All mosquitoes were 100% susceptible to pyrethroids, DDT, bendiocarb and fenitrothion. To measure the ability of the EO to kill adult mosquitoes, bottle test bioassays were conducted according to the method of Brogdon and McAllister [30], with acetone as solvent. Twenty-five mosquitoes, aged three days from the susceptible Dakar strain, were used. For each EO, solutions were prepared at concentrations of 8.3 μg/ml, 16.6 μg/ml, 24.91 μg/ml, 32.22 μg/ml, 41.52 μg/ml, 49.88 μg/ml, 58.13 μg/ml and 66.44 μg/ml. Four replicates were set up for each concentration, using 1 ml of solution/bottle. Deltamethrin 12.5 μg/ml was used for positive control while the negative control was acetone. Mortality was assessed every 15 min. With practice, the mortality of mosquitoes in the control bottle at two hours should be zero. In most cases, mortality of up to 3% in the control bottles may be ignored. The data were corrected using the Abbot’s formula [31] for mortalities in the control ranging 3-10%. A dose-mortality line depending on the exposure time was developed.

### Larvicidal activity

The larvicidal activity was performed according to the guidelines for laboratory and field testing of mosquito larvicides published by WHO (who/cds/whopes/gcdpp/2005.13), with minor modifications.

A laboratory colony of *An. gambiae* larvae (susceptible strain) was used for the larvicidal activity of EO collected at the three stages (samples A, B and C). Twenty-five third instars larvae were kept in 500 ml glass beaker containing aqueous suspension of EO at dilution from 14 to 75%. Four replicates were set up for each dilution. The negative control was exposed to water. Larval mortality was assessed after 24 hrs of exposure by probing the larvae with needle and moribund larvae were counted as dead. A dose-mortality line was recorded and the lethal concentration of EO needed to kill 50% (LC_50_) of larvae was determined.

### Statistical analysis

Statistical analyses were performed with Graph Pad Prism 4 software, using t test or one-way analysis of variance followed by Bonferroni’s post hoc test. The significance was set at p <0.05.

## Results

### Essential oil yield and composition

The yield of the EOs from *S. thymbraM. communis* and *T. herba-barona* collected before (A), during (B) and after (C) flowering are reported in Table 1. Maximum amount of the oil was present during flowering, with the exception of *T. herba-barona*. Tables 2,3, and 4 list the components identified in the EOs, together with the Linear Retention Indices (*I*_s_^T^) on Mega 5 column compared to the corresponding value from the literature [24], and the quantitative data (g/100 g) obtained by using the response factors (RFs) calculated for all the chemical groups present in the EO (see Methods). The EO of *M. communis* was rich in α-pinene, limonene and 1,8-cineole (Table 2), the EO of *T. herba-barona* was rich in linalool and carvacrol (Table 3), the EO of *S. thymbra* was rich in thymol and γ-terpinene (Table 4). Since the EO of *S. thymbra* was the most active against *P. falciparum*, it was submitted to fractionation. Three main fractions (1–3) were obtained from each *S. thymbra* EO (Table 5): fraction A1, B1 and C1 are characterized by a mixture of monoterpene and sesquiterpene hydrocarbons accounting respectively for the 47%, 48% and 20% of the total oil; in fractions A2, B2, C2 thymol and carvacrol are the main components accounting for about 80% of the composition of these fractions representing 8%, 24% and 33% of the total oil, respectively; in fractions A3, B3, and C3 which represent only 7% of the total oil, thymol content is lower with respect to the corresponding fraction 2.

**Table 1:** Collection date and site, and EO recovery

**Plant**	Sample*	**Collection date**	**Site**	**Drug weight (Kg)**	**Oil weight (g)**	**Oil volume (ml)**
	A	April 2009		3.4	2.0	3.0
*M. communis* aerial parts	B	June 2009	Jerzu (Ogliastra)	1.5	6.8	8.5
	C	July 2009		2.6	5.5	6.5
	A	April 2009		2.0	5.4	6.0
*S. thymbra* aerial parts	B	June 2009	Cagliari	2.3	15.7	17.0
	C	July 2009		1.1	8.0	9.3
	A	May 2009		1.0	3.0	3.5
*T. herba-barona* aerial parts	B	June 2009	Osini (Ogliastra)	0.8	1.7	2.2
	C	July 2009		1.4	4.9	6.0

* sample A: before flowering; sample B: during flowering; sample C: after flowering.

**Table 2:** **Composition of*Myrtus communis*essential oil before (A), during (B) and after (C) flowering**

			**Sample A**	**Sample B**	**Sample C**
**Compound**	***I***^**T**^_**S**_**.EXP**^**a**^	***I***^**T**^_**S**_**. LIT**^**b**^	**g/100g**	**g/100g**	**g/100g**
α-Thujene	929	930	0.56	0.75	0.69
**α-Pinene**	**938**	**936**	**78.00**	**56.6**	**54.77**
Sabinene	975	975	n.r.	0.12	0.07
β-Pinene	977	979	0.45	0.60	0.58
β-Myrcene	992	991	0.12	0.31	0.20
α-Phellandrene	1003	1003	0.35	1.34	0.64
δ-2-Carene	1009	1002	0.84	0.98	0.94
α-Terpinene	1017	1017	0.07	0.35	0.19
*p*-Cimene	1025	1025	1.32	0.51	1.27
**Limonene**	**1030**	**1029**	**5.54**	**5.68**	**6.53**
*E*-β-Ocimene	1051	1050	0.37	1.06	0.78
γ-Terpinene	1060	1060	0.53	1.47	1.03
α-Terpinolene	1088	1089	0.50	1.86	1.20
Linalool	1099	1097	0.66	1.55	1.85
Terpinen-4-ol	1177	1177	0.07	0.27	0.34
α-Terpineol	1189	n.r.-	0.32	3.00	3.40
Geraniol	1257	1253	0.11	0.57	0.32
Terpinyl acetate	1350	1349	0.12	0.57	n.r.
Eugenol	1357	1359	n.r.	0.15	n.r.
Geranyl acetate	1385	1381	1.16	2.38	2.23
β-Elemene	1390	1391	0.12	0.08	0.41
Methyl eugenol	1404	1404	0.25	0.41	0.33
β-Caryophyllene	1416	1419	1.58	1.41	1.23
γ-Elemene	1432	1437	0.10	0.39	0.19
α-Humulene	1451	1455	0.54	0.47	0.44
β-Selinene	1483	1490	n.r.	n.r.	0.17
α-Selinene	1491	1498	n.r.	n.r.	0.21
δ-Cadinene	1521	1523	0.10	0.10	0.11
Germacrene B	1554	1561	0.28	0.97	0.40
Caryophyllene oxide	1579	1583	1.11	0.10	0.25
TOTAL			99.32	99.64	99.70

GC-FID-MS analyses were carried out on a Mega5 column (5%phenyl methyl polysiloxane) 25 m, 0.25 mm i.d., 0.25 μm film thickness.

^a^Experimental Linear Retention Index on Mega5 Column.

^b^ Linear Retention Index on Mega5 Column from Literature (Ref Adams 2007).

**Table 3:** **Composition of*Thymus herba-barona*essential oil before (A) during(B) and after (C) flowering**

			**Sample A**	**Sample B**	**Sample C**
**Compound**	***I***^**T**^_**S**_**.EXP**^**a**^	***I***^**T**^_**S**_**.LIT**^**b**^	**g/100g**	**g/100g**	**g/100g**
Heptanone	-	892	1.30	1.61	1.81
α-Thujene	929	930	0.17	0.18	0.16
α-Pinene	935	936	0.17	0.25	0.19
Camphene	950	954	0.25	0.43	0.33
Octanone	987	984	2.03	2.38	2.32
β-Myrcene	992	991	0.24	0.23	0.20
Octanol	996	995	0.20	0.98	1.07
α-Terpinene	1017	1017	0.20	0.19	0.19
*p*-Cymene	1025	1025	1.00	0.87	0.94
Limonene	1030	1029	0.11	0.10	0.11
γ-Terpinene	1060	1060	1.83	1.45	1.24
*Z*-Sabinene hydrate	1068	1070	0.23	0.25	0.09
Nonanone	1088	1090	0.41	0.46	0.59
**Linalool**	**1104**	**1097**	**38.86**	**47.26**	**55.15**
Borneol	1166	1169	2.89	3.63	3.05
Terpinen-4-ol	1177	1177	0.44	0.44	0.45
Carvacrol methyl ether	1245	1245	0.27	1.38	0.55
Thymol	1293	1290	0.81	0.70	0.22
**Carvacrol**	**1307**	**1299**	**45.00**	**34.60**	**28.59**
Carvacrol acetate	1374	1373	0.31	n.r.	n.r.
β-Caryophyllene	1417	1419	2.20	2.06	1.67
trans-α-Bergamotene	1435	1435	0.06	0.06	0.06
α-Humulene	1451	1455	0.08	0.08	0.06
Caryophyllene oxide	1579	1583	0.63	0.32	0.38
TOTAL			99.69	99.91	99.42

^a^ Experimental Linear Retention Index on Mega5 Column.

^b^ Linear Retention Index on Mega5 Column from Literature (Adams 2007).

**Table 4:** **Composition of*Satureja thymbra*essential oil before (A) during (B) and after (C) flowering**

			**Sample A**	**Sample B**	**SampleC**
**Compound**	***I***^**T**^_**S**_**EXP**^**a**^	***I***^**T**^_**S**_**. LIT**^**b**^	**g/100g**	**g/100g**	**g/100g**
*α*-Thujene	929	930	1.1	1.1	0.9
*α*-Pinene	935	936	1.5	1.4	1.7
Camphene	950	954	0.7	0.6	0.7
Sabinene	974	975	0.2	0.2	0.1
*β*-Pinene	977	979	0.7	0.7	0.9
*β*-Myrcene	991	991	1.4	1.2	1.2
*α*-Phellandrene	1003	1003	0.2	0.2	0.2
*α*-Terpinene	1017	1017	2.4	2.0	1.6
*p*-Cymene	1025	1025	6.7	4.3	4.8
Limonene	1029	1029	0.8	0.6	0.7
*Cis β*-Ocimene	1040	1037	1.0	0.3	0.3
*Trans β*-Ocimene	1050	1050	1.2	0.4	0.4
***γ*-Terpinene**	**1061**	**1060**	**27.0**	**18.2**	**11.8**
cis-Sabinene hydrate	1068	1070	0.1	0.1	0.2
*α*-Terpinolene	1088	1089	0.1	0.1	0.1
trans-Sabinene hydrate	1097	1098	0.1	0.1	0.1
Linalool	1099	1097	0.1	0.2	0.2
Borneol	1165	1169	1.0	1.0	1.6
Terpinen-4-ol	1177	1177	0.3	0.3	0.4
Thymol methyl ether	1244	1235	1.8	1.3	1.8
**Thymol**	**1293**	**1290**	**36.0**	**52.6**	**57.1**
Carvacrol	1300	1299	1.8	2.8	3.5
Thymol acetate	1354	1352	0.2	0.1	0.3
*β*-Caryophyillene	1417	1419	5.6	5.0	5.1
*α*-Humulene	1451	1455	0.2	0.2	0.2
Germacrene D	1479	1485	0.5	0.4	0.1
Caryophyllene oxide	1579	1583	0.9	0.3	0.6
TOTAL			93.6	95.7	96.5

^a^ Experimental Linear Retention Index on Mega5 Column.

^b^ Linear Retention Index on Mega5 Column from Literature (Ref Adams 2007).

**Table 5:** **Seasonal composition of*S. thymbra*EO after fractionation**

**Compounds**	% Composition*								
	**A1**	**A2**	**A3**	**B1**	**B2**	**B3**	**C1**	**C2**	**C3**
γ-terpinene	54			50			39		
β-caryophyllene	15			15			32		
*p*-cymene	10			11			12		
thymol-methyl-ether	4			-	9		-	10	
**thymol**		**83**	**61**		**75**	**51**		**73**	**47**
carvacrol		5	21		11	25		11	22
thymol acetate		3			0.5			1	
caryophyllene oxide		8			3			3	
terpinen-4-ol			5			7			8
Thym./carv. ratio	-	16.6	2.9	-	6.8	2.0	-	6.6	2.1

* sample A: before flowering; sample B: during flowering; sample C: after flowering.

Fraction 1-3 were obtained through fractionation on silicagel column chromatography and analysed by CG-FID-MS on a Mega5 column (5%phenyl methyl polysiloxane) 25 m, 0.25 mm i.d., 0.25 μm film thickness.

### Anti-plasmodial activity

The anti-plasmodial activity of the EOs of *S. thymbra*, *M. communis* and *T. herba-barona* is reported in Table 6. All EOs inhibited the growth of CQ-resistant and CQ-sensitive strains of *P. falciparum* in a dose-dependent manner. The EO of *S. thymbra* was the most active compared to the EOs of the other plants: the anti-plasmodial effect of the EOs collected at the three different flowering stages was in a similar range of IC_50_s, namely 9–11 μg/ml against D10, and 17.5-26.1 μg/ml against W2.

**Table 6:** **Antiplasmodial activity of the essential oils (EO) of*S. thymbra*,*M. communis*and*T. herba-barona***

**EO**	**D10 IC**_**50**_**(μg/ml)**	**W2 IC**_**50**_**(μg/ml)**
*M. communis* A	37.3 ± 3.5	28.5 ± 8.9
*M. communis* B	>50.0	36.5 ± 10.3
*M. communis* C	21.8 ± 2.2	18.3 ± 12.5
*S. thymbra* A	26.1 ± 10	11.3 ± 6.5
*S. thymbra* B	17.5 ± 6.6	9.0 ± 3.3
*S. thymbra* C	21.3 ± 4.2	9.7 ± 2.8
*T. herba-barona* A	29.1 ± 6.6	26.2 ± 2.2
*T. herba-barona* B	29.4 ± 4.9	24.2 ± 7.6
*T. herba-barona* C	>50.0	28.8 ± 12.2
CQ^a^	0.007 ± 0.0001	0.11 ± 0.04

^a^CQ: chloroquine; results are the mean ± s.d. of three experiments in duplicate.

A) before flowering; B) during flowering; C) after flowering.

*S. thymbra* EO at the flowering stage (sample B) was tested for inhibition of plasmepsin II and the IC_50_ was 29.5 ± 0.09 μg/ml (mean ± sd). The potency for killing Plasmodium and for inhibition of the enzyme was comparable, thus indicating that plasmepsin II may be one of the molecular targets of its pharmacological effect.

**Figure 1:**
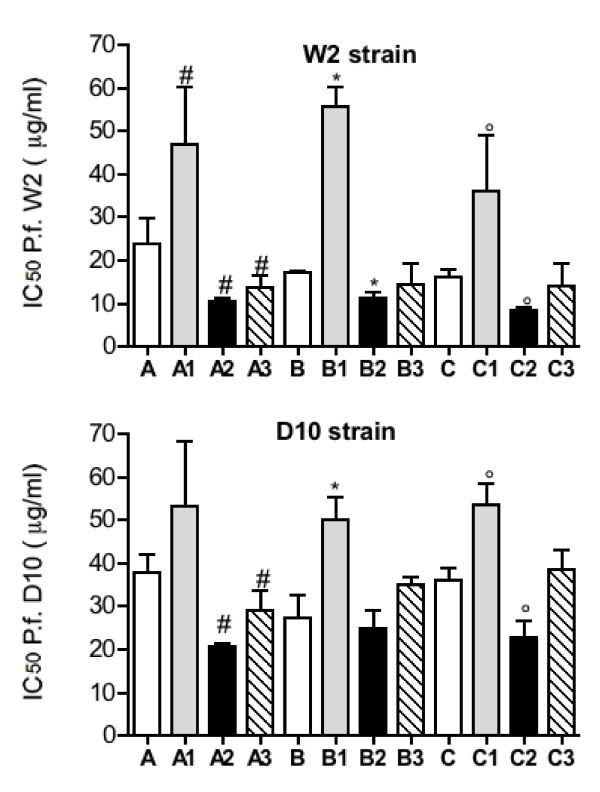
**Anti-plasmodial activity of fractions 1–3 of*Satureja thymbra*EO.** Fractions were dissolved in DMSO and then diluted with a medium to achieve the required concentrations (final DMSO concentration <1%, which is non-toxic to the parasite). Asynchronous cultures with parasitaemia of 1–1.5% and 1% final haematocrit were aliquoted into the plates and incubated for 72 hrs at 37°C. Parasite growth was determined spectrophotometrically (OD650) by measuring the activity of the parasite lactate dehydrogenase (LDH). IC_50_ is the mean ± standard deviation of three separate experiments performed in duplicate. # statistically different *vs* A; * statistically different *vs* B; ° statistically different *vs* C. CQ was used as reference compound (IC_50_ 7.0 and 110 ng/ml on CQ-S and CQ-R strains, respectively).

The effect of fractions 1–3 obtained from the EO of *S. thymbra* is reported in Figure 1. The highest inhibition of parasite growth was observed using the fractions A2, B2 and C2 where thymol is the main component. Fractions A1, B1 and C1, where thymol was absent, showed a very low effect. The anti-plasmodial effect of thymol (IC_50_ 19.7 ± 3.0 and 10.6 ± 2.0 μg/ml on D10 and W2, respectively) confirmed that this compound is the active principle. The W2 strain rather than D10 seems to be more susceptible to the effects of the EO sub fractions and thymol.

### Larvicidal and adulticidal activity against *anopheles gambiae*

**Figure 2:**
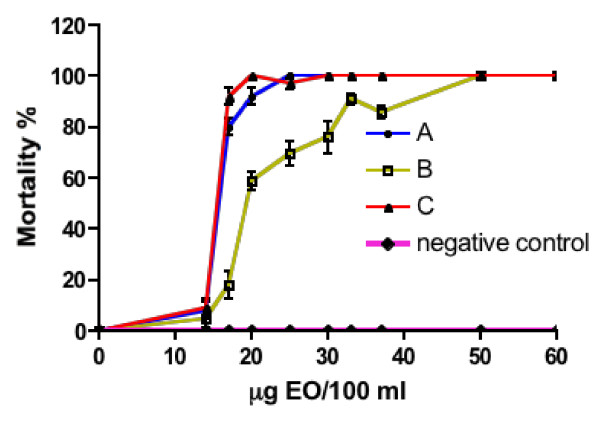
**Evolution of the larvicidal activity at different dilutions of*Satureja thymbra*EO.** Twenty-five third instar larvae of a laboratory colony of *Anopheles gambiae* (susceptible strain) were used. Mortality % was assessed at increasing concentration of EOs collected before, during, and after flowering. Four replicates were set up for each concentration. Water was used as negative control. The activity of the EOs expressed as LC_50_ was 0.15 ± 0.002; 0.21 ± 0.013; and 0.15 ± 0.087 μg/ml (mean ± sd) for the samples A, B and C, respectively.

**Figure 3:**
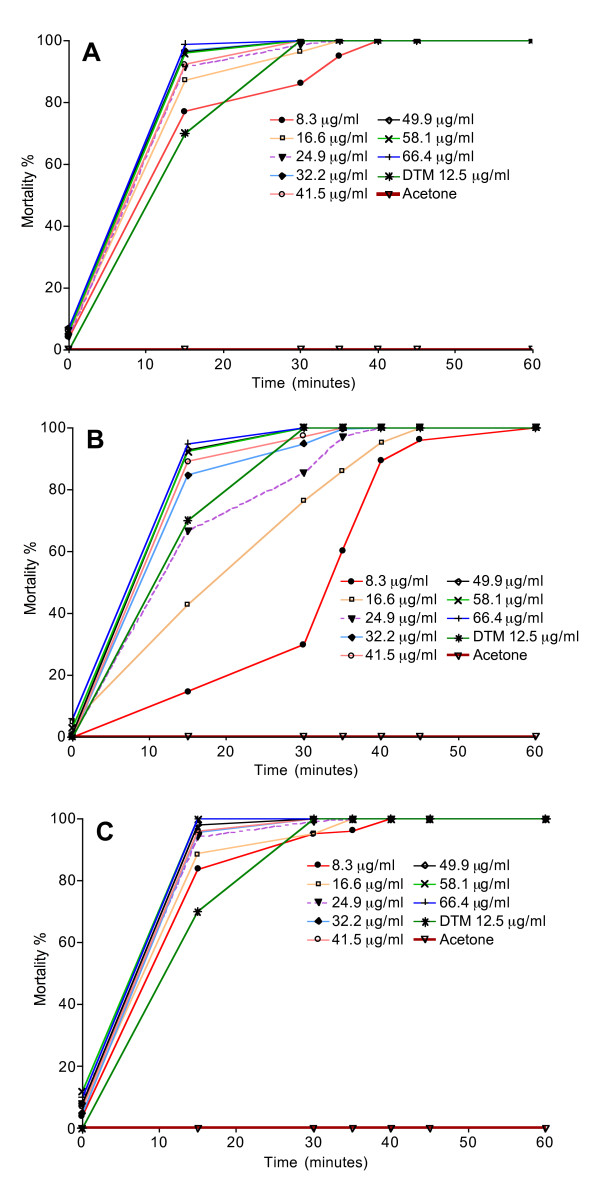
**Time-course of the adulticidal activity of*Satureja thymbra*EO.** Twenty-five mosquitoes aged three days from the susceptible Dakar strain were used. For each EO, solutions were prepared in acetone at concentrations of 8.3 μg/ml, 16.6 μg/ml, 24.91 μg/ml, 32.22 μg/ml, 41.52 μg/ml, 49.88 μg/ml, 58.13 μg/ml and 66.44 μg/ml. Four replicates were set up for each concentration, using 1 ml of solution/bottle. Deltamethrin 12.5 μg/ml was used for positive control while the negative control was acetone. Mortality was assessed every 15 min. **A**) before flowering; **B**) during flowering; **C**) after flowering.

The mortality rate of the larvae of *An. gambiae* increased dose-dependently in the presence of EOs of *S. thymbra* (Figure 2). The activity of the EO expressed as LC_50_ was 0.15 ± 0.002; 0.21 ± 0.013; and 0.15 ± 0.087 μg/ml (mean ± sd) for the samples A, B and C, respectively. Samples A and C were significantly different *versus* sample B (p < 0.002). The larvicidal activity was dependent on the composition of EO being higher for the EO collected before and after flowering, compared to those collected during flowering. The time-course of the adulticidal activity is shown in Figure 3. Following the same trend as the larvicidal effect, the insecticidal activity against the adult stage of the mosquito is greater when the EO was obtained from plant material collected before and after flowering. At the lowest concentration tested (8.3 μg/ml), 100% mosquitoes died within 40 min when exposed to sample A and C. For sample B the time of exposure required to kill 100% of insects was 60 min.

### Cytotoxicity on human dermal fibroblasts

The results are reported in Table 7. The EO of *S. thymbra* (samples A, B, C) and sub-fractions A1-A2, B1-B2, and C1-C2 in the range of 10–50 μg/ml were not toxic in human fibroblasts at 24 hrs of exposure. By extending the time of exposure to 48 hrs, cell viability was either not different from untreated cells (A1, B, B1, C) or reduced (A, A2, B2, C1, C2). Sub-fractions A3, B3 and C3 caused 100% mortality at 50 μg/ml independently of the time of exposure. These fractions are characterized by higher levels of carvacrol with respect to fraction 1 and 2, indicating that carvacrol may be responsible for cytotoxicity. The exposure of fibroblasts to 100 μg/ml of EO and sub-fractions caused a rise of cell mortality. In general with longer time of exposure, cell viability diminished. Thymol at 50 μg/ml (five-fold higher than the IC_50_ on *P. falciparum*) for 24 hrs did not influence cell viability, but became more toxic at 48 hrs (50% reduction of cell viability). It is worthy to underline that the concentration of EO causing toxicity to human cells (100 μg/ml) are far and best higher than those required for the larvicidal or mosquitocidal effects (0.15 and 8 μg/ml, respectively).

**Table 7:** **Cytotoxicity of the EO of*Satureja Thymbra*L**

	**24 hrs**			**48 hrs**		
**Sample**	**Concentration (μg/ml)**					
	**25**	**50**	**100**	**25**	**50**	**100**
**A**	NT	NT	15.3	NT	12.3	24.4
**A1**	NT	NT	NT	NT	NT	33.0
**A2**	NT	NT	NT	NT	9.0	94.7
**A3**	15	100	100	100	100	100
**B**	NT	NT	12.2	NT	NT	13.3
**B1**	NT	NT	NT	NT	NT	24.0
**B2**	NT	NT	24.3	19.7	47.0	94.0
**B3**	18.0	100	100	100	100	100
**C**	NT	NT	14.3	NT	NT	22.2
**C1**	NT	NT	NT	NT	35.3	85.2
**C2**	NT	NT	24.4	14.0	31.6	95.3
**C3**	32	100	100	100	100	100

Data are reported as % mortality vs ctrl (0 %). NT: not toxic (viability not statistically different with respect to ctrl). A) before flowering; B) during flowering; C) after flowering.

## Discussion

Aromatic plants are frequently investigated as a possible source of antimicrobial/insecticide compounds. In the past, the Sardinian population traditionally employed local aromatic plants against malaria.

Among the plants of the Sardinian flora, myrtle, savory, and caraway thyme have an historical witness of use and thus were chosen for this study. Among the three plants, *S. thymbra* showed the highest *in vitro* anti-plasmodial activity, especially against the CQ-resistant strain. Moreover, the time of collection (before, during, and after flowering) did not influence the anti-plasmodial effect, which appeared to be mainly associated with thymol, one of the components of the EO. These results are in agreement with a recent report that the EO of *Oreganum compactum*, rich in thymol, shows anti-plasmodial activity *in vitro*[32]. Such a conclusion is corroborated by the data obtained with the EOs of *M. communis* and *T. herba-barona* since the latter, which does not contain thymol, possesses lower activity against *P. falciparum.* Moreover, the activity of thymol was selective against the parasites with low cytotoxicity against human dermal fibroblasts.

The anti-plasmodial activity of the EOs of various aromatic plants has been investigated [5,32-34], however the mechanism of action still remains obscure. When tested on a highly synchronized culture, the EO of *Lippia multiflora* inhibited the growth mostly at the trophozoite-schizont step, indicating a potential effect on the first nuclear division of the parasite [33]. In the present work savory EO was tested against plasmepsin II and caused enzyme inhibition at a concentration very close to that needed for killing the parasite. Even if preliminary this observation suggests the food vacuole as the site of action and plasmepsins as one of the possible targets. The absence or very low toxicity on human cell lines indicates a selectivity of savory EO for targets specific of *P.falciparum.* In the present work, the bioassays were performed on the erythrocytic stage of the parasite, then the molecular targets are to be searched within those operating at this point of the life cycle.

Malaria control and elimination will not be achieved without the control of transmission through the use of repellents/insecticides against the mosquito vector. The vector control is among the key strategies promoted by WHO and The Roll Back Malaria Partnership for prevention, control and elimination of malaria [35]. Insecticide-treated nets and indoor residual spraying are effective methods for malaria vector control. Larval control at the breeding sites can be used complementary to other vector control methods [36].

A large number of phytochemicals including volatile oils exhibited larvicidal and mosquitocidal activity [37]. In this regard, it is interesting that the EO of *S. thymbra* is highly active against both the larvae and the adults of *Anopheles* mosquitoes. The vegetative period produced EO with different composition, probably not affecting the efficacy of the insecticidal bio-activity. Even if EOs obtained from savory at the different vegetative stages show statistically significant differences, these differences are minimal, and thus not relevant in term of efficacy for pest control.

In consideration of the toxicity tests on human cells, and the dose at which savory EO is active as larvicidal (IC_50_ 0.15–0.2 μg/ml), the use of savory for vector control is rather promising and deserves further investigation in order to test individual prominent components and verify whether the mixture is less or more potent than the compounds alone. Synergistic or antagonistic phenomena have been described at this regard [11,38].

The mode of action and site of effect for larvicidal/mosquitocidal phytochemicals have received little attention so far. Hypotheses for mechanistic action can be neurotoxicity, growth regulation, endogenous hormone agonist or antagonist [11,38]. The investigation about the mechanism of action of EO would be helpful for development of natural insecticides with low toxicity and with little impact on the environment.

## Conclusions

The use of aromatic plants as a source of insecticides and/or repellents is a traditional practice still in wide use throughout developing countries [3]. The inhalation of plants containing essential oils as febrifuge in malaria remains nowadays a common practice in traditional medicine in West African countries, such as Cameroon, where the population inhales the vapours of a decoction of *Cymbogon citratus* and *Ocimum gratissimum* to treat fevers due to malaria [5].

This study provides evidence for the use of EOs for treating malaria and fighting the vectors at both larval and adult stages, thus supporting the Sardinian traditional use of savory against malaria. The research reported herein opens the possibility for further investigation and isolation of natural products with anti-parasitic properties in order to overcome the problem of insecticide resistance.

## Abbreviations

EO, Essential oil; FID, Flame ionization detector; PE, Petroleum ether; GC, Gas chromatography; EtOAc, Ethyl acetate; MS, Mass spectrometry; CQ, Chloroquine; DMSO, Dimetilsulfoxide; LDH, Lactate dehydrogenase; EI, Ionization mode; PLM, Plasmepsin; MTT, (3-[4,5-dimethylthiazol-2-yl]-2,5-diphenyltetrazolium bromide); sd, Standard deviation.

## Competing interest

The authors declare that they have no competing interests.

## Authors’ contributions

MDA, CS and MB designed the research. CS and MB supplied the plant material and EOs. EC, NB, MMS and DT performed the experiments for the anti-plasmodial activity. PT, LM and CB performed the analyses of EO composition. MON was responsible for the larvicidal and insecticidal experiments. MDA and EB drafted and wrote the final manuscript. All authors approved the final manuscript.
